# Structure of the Human FANCL RING-Ube2T Complex Reveals Determinants of Cognate E3-E2 Selection

**DOI:** 10.1016/j.str.2013.12.004

**Published:** 2014-02-04

**Authors:** Charlotte Hodson, Andrew Purkiss, Jennifer Anne Miles, Helen Walden

**Affiliations:** 1 Protein Structure and Function Laboratory, Lincoln’s Inn Fields Laboratories of the London Research Institute, Cancer Research UK, 44 Lincoln’s Inn Fields, London WC2A 3LY, UK; 2 MRC-Protein Phosphorylation and Ubiquitylation Unit, College of Life Sciences, Dow Street, Dundee DD1 5EH, UK

## Abstract

The combination of an E2 ubiquitin-conjugating enzyme with an E3 ubiquitin-ligase is essential for ubiquitin modification of a substrate. Moreover, the pairing dictates both the substrate choice and the modification type. The molecular details of generic E3-E2 interactions are well established. Nevertheless, the determinants of selective, specific E3-E2 recognition are not understood. There are ∼40 E2s and ∼600 E3s giving rise to a possible ∼24,000 E3-E2 pairs. Using the Fanconi Anemia pathway exclusive E3-E2 pair, FANCL-Ube2T, we report the atomic structure of the FANCL RING-Ube2T complex, revealing a specific and extensive network of additional electrostatic and hydrophobic interactions. Furthermore, we show that these specific interactions are required for selection of Ube2T over other E2s by FANCL.

## Highlights

• Structure of the ubiquitination platform FANCL-Ube2T is essential for DNA ICL repair
• Structural basis for cognate E2-E3 enzyme pairing
• Interactions beyond the generic E2-E3 interface are required for E2-E3 selectivity

## Graphical Abstract

**Figure:**
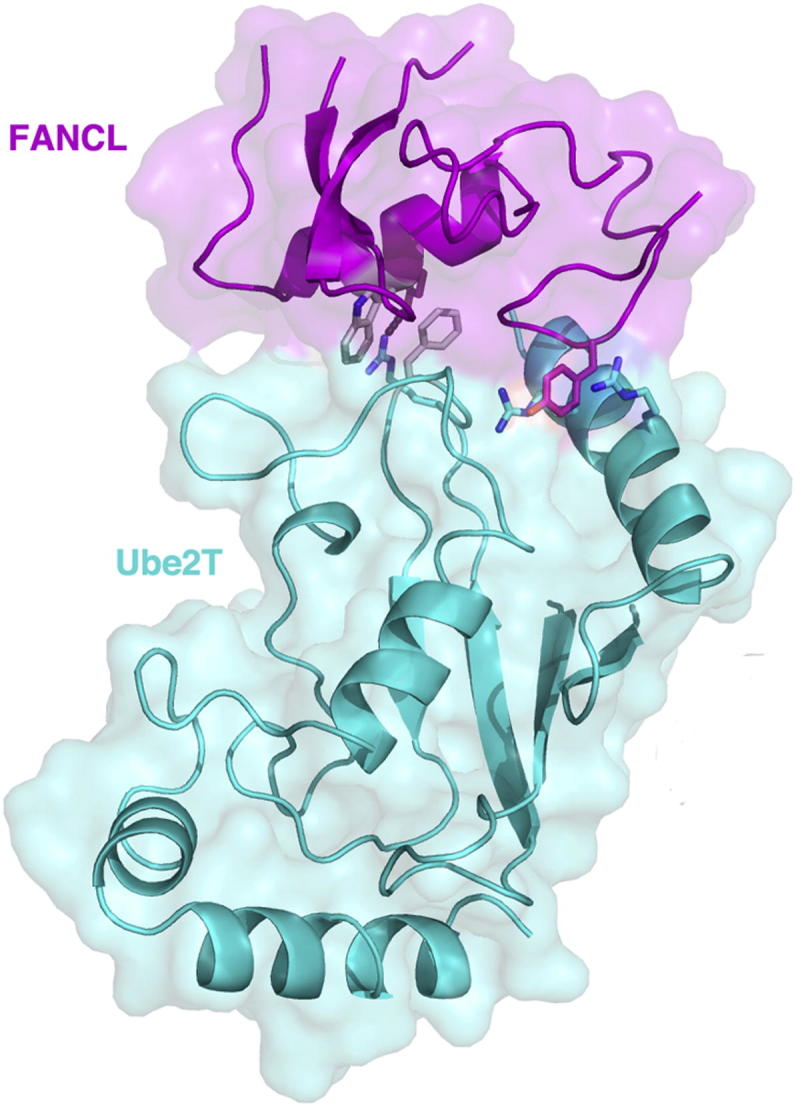

## Teaser

Ube2T and FANCL represent a specific E2-E3 ubiquitin-conjugating enzyme and ubiquitin ligase pair required for the function of the Fanconi anemia DNA repair pathway. Hodson et al. report a structure of Ube2T in complex with the RING domain of FANCL and reveal molecular determinants of E2-E3 specificity.

## Introduction

Ubiquitination is a reversible posttranslational modification in which ubiquitin (Ub) is covalently attached via its C terminus, typically to a substrate lysine. Ubiquitination is required for the strict regulation of a wide range of essential cellular processes, from protein degradation to DNA repair and cell-cycle control (Pickart and Eddins, 2004). Consequently, defects that arise in the regulation of ubiquitination can lead to a variety of diseases, such as cancers and neurodegeneration.

Substrate ubiquitination is achieved through an enzyme cascade involving an E1 activating enzyme, an E2 ubiquitin-conjugating (UBC) enzyme, and an E3 ligase. The E3 ligase, in combination with its partnered E2 enzyme, coordinates the transfer of ubiquitin onto a specific lysine residue. The E3-E2 pair also dictates the type of modification, ranging from monoubiquitination to Ub polymers (Ye and Rape, 2009). The human genome encodes two E1 enzymes, approximately 40 E2s and over 600 E3 ligases, giving rise to thousands of possible permutations of E3-E2 pairings.

Experimentally determined structures of E3-E2 complexes have revealed a well-conserved hydrophobic interaction surface, encompassing loops 1 and 2 and the first helix of the E2. (Bentley et al., 2011; Dou et al., 2012; Huang et al., 1999; Plechanovová et al., 2012; Pruneda et al., 2012; Yin et al., 2009; Zheng et al., 2000). Furthermore, the conservation of the E2 UBC fold, along with the conservation of the hydrophobic residues for the E3-interacting interface, suggests that all E3s could function with all E2s (van Wijk and Timmers, 2010). Yet, this is not what is observed in nature, as there is selectivity in E3-E2 pairs with some pairs being exclusive (Bailly et al., 1994; Chen et al., 2006). There have been great efforts using yeast-two-hybrid screens, computational biology, and modeling methods to determine E3-E2 pairs (Kar et al., 2012; Markson et al., 2009; van Wijk et al., 2009). A recent proteome scale modeling study, aimed at identifying determinants of E3-E2 specificity, predicts residues on loop 1 of the E2 to be important for E3 selection (Kar et al., 2012). Additionally, there is much interest in creating new E3-E2 pairs or enhancing specificity (Starita et al., 2013; Winkler and Timmers, 2005), both for understanding ubiquitin biology and from a therapeutic perspective. However, these aims are hampered by the lack of molecular details and structural data as to what constitutes a specific E3-E2 pair.

An example of an exclusive E3-E2 pair is the catalytic center of the Fanconi Anemia (FA) pathway, FANCL-Ube2T (Alpi et al., 2008; Machida et al., 2006). The FA pathway is required for DNA interstrand crosslink repair. Mutations in the FA pathway result in the genetic disorder known as Fanconi Anemia, where patients have high predispositions to cancers because of their genomic instabilities (Alter, 1996). FANCL is a monomeric RING E3 ligase (Cole et al., 2010; Meetei et al., 2003), which specifically interacts with the E2, Ube2T (Alpi et al., 2008; Machida et al., 2006), for the strict monoubiquitination of FANCD2 (Garcia-Higuera et al., 2001; Timmers et al., 2001). This monoubiquitination event is key in signaling the recruitment of downstream DNA repair factors (Kottemann and Smogorzewska, 2013).

## Results and Discussion

**Figure 1:**
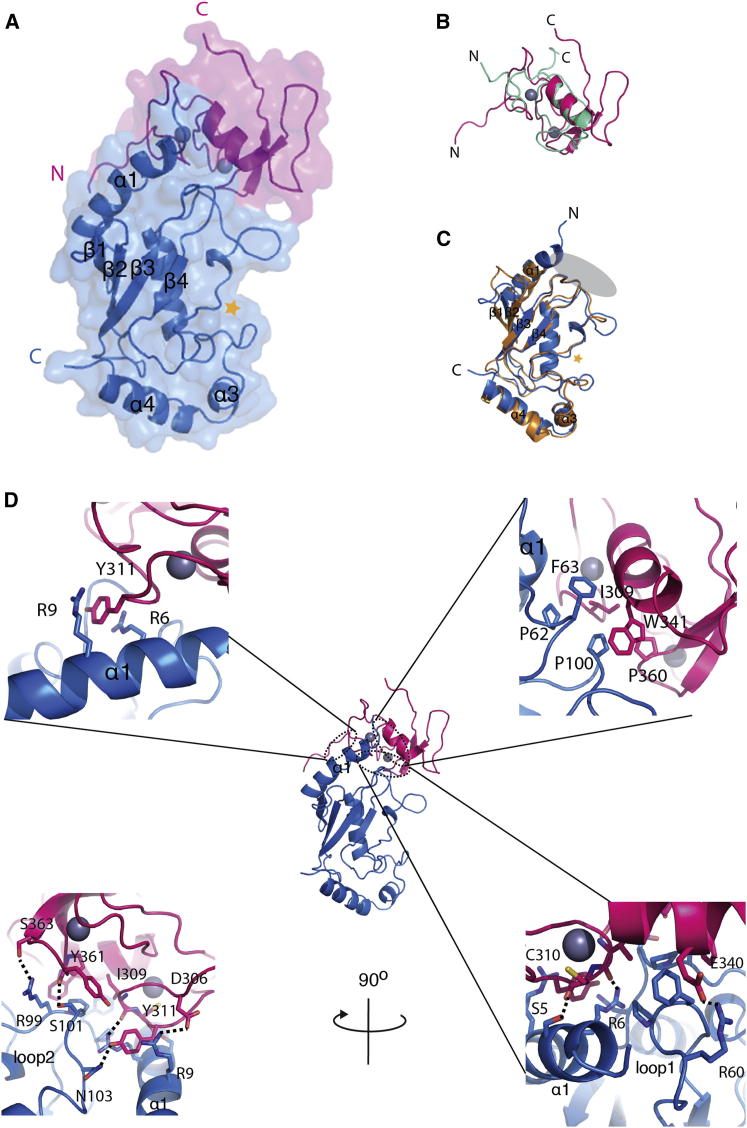
Overall Structure of FANCL-Ube2T Complex (A) The overall structure of the RING domain of FANCL (magenta) bound to Ube2T (blue) is shown in cartoon representation. Gray spheres represent zinc ions. A gold star represents the position of Ube2T’s catalytic cysteine. (B) RING domain of FANCL (magenta) overlain with c-cbl RING domain (green; PDB ID code 1FBV). (C) Ube2T (blue) overlain with Ube2L3 (orange; PDB ID code 1FBV) showing the structural conservation of the UBC fold, comprising a four-stranded β-meander flanked by an N-terminal helix (helix1) and two C-terminal helices (helixes 2 and 3). A gold star represents the position of the catalytic cysteine. The gray oval shows the E3 binding interface of E2s. (D) Top left panel: The pi stacking in the binding interface between Y311 of FANCL and R6 and R9 of Ube2T. Top right panel: The hydrophobic binding interface of the RING domain (magenta) and Ube2T (blue). Bottom panels: The electrostatic and hydrogen bonding network of the RING-Ube2T interface. Interactions are represented by dashed lines. See also Figure S1.

FANCL and Ube2T coelute as a 1:1 stoichiometric complex by size-exclusion chromatography and have an affinity with a dissociation constant (K_D_) of ∼0.5 μM (Hodson et al., 2011). The complex crystallizes with a diffraction limit of ∼11 Å. In order to obtain high-resolution data to observe the interface, we fused human Ube2T to the C terminus of the human FANCL RING domain with a linker between the proteins. Subsequently, we determined the structure for the E3-E2 pair, the FANCL RING domain (residues 299–373), and Ube2T (residues 1–153) to 2.4 Å resolution (Figure 1A; Table 1).

The human RING domain contains two zinc atoms coordinated by a (Cys)_4_, His, (Cys)_3_ arrangement in a cross-brace structure. The arrangement of cysteine and histidines differs slightly to the (Cys)_3_, His, (Cys)_4_ arrangement observed in other RING domains. This unusual arrangement is also noted in the *Drosophila* FANCL structure (Cole et al., 2010) and is conserved across all other FANCL homologs. An overlay with the *Drosophila* FANCL RING domain reveals that the homologs are highly similar with a root-mean-squared deviation (rmsd) of 1.7 Å across all alpha-carbon atoms (Figure S1A available online). In common with other RING domains, FANCL contains the helical element involved in E2 recognition (Figure 1B) (Deshaies and Joazeiro, 2009). In complex with FANCL, Ube2T adopts a typical UBC-fold comprising a four-stranded beta meander, flanked by an N-terminal helix and two C-terminal helices (Figure 1C). In order to determine whether significant conformational changes occur in Ube2T upon RING binding, we superimposed the bound Ube2T in our structure to unbound Ube2T (Protein Data Bank [PDB] ID code 1YH2) (Sheng et al., 2012). The two structures align with an rmsd of 0.67 Å across all alpha-carbon atoms, indicating that no major structural rearrangements occur upon complex formation (Figure S1B).

The interface between the RING domain and Ube2T buries a total surface area of ∼700 Å^2^. In common with other E3-E2 structures, the interface consists of a conserved hydrophobic interface between Pro62, Phe63, and Pro100 of Ube2T and Ile309, Trp341, and Pro360 of FANCL (Figure 1D), as observed in other RING-E2 structures (Bentley et al., 2011; Dou et al., 2012; Plechanovová et al., 2012; Pruneda et al., 2012; Yin et al., 2009; Zheng et al., 2000). However, the hydrophobic surface of FANCL is extended by Tyr311, which is involved in pi stacking between Arg6 and Arg9 (Figure 1D). Further analysis of the interface reveals an extensive electrostatic and hydrogen bonding network between residues Ser5, Arg6, Arg9, Arg60, Arg99, Ser101, and Asn103 of Ube2T and Asp306, Tyr311, Glu340, and Ser363 of FANCL, with additional main-chain interactions with Ile309, Cys310, and Tyr361 of FANCL (Figure 1D).

**Figure 2:**
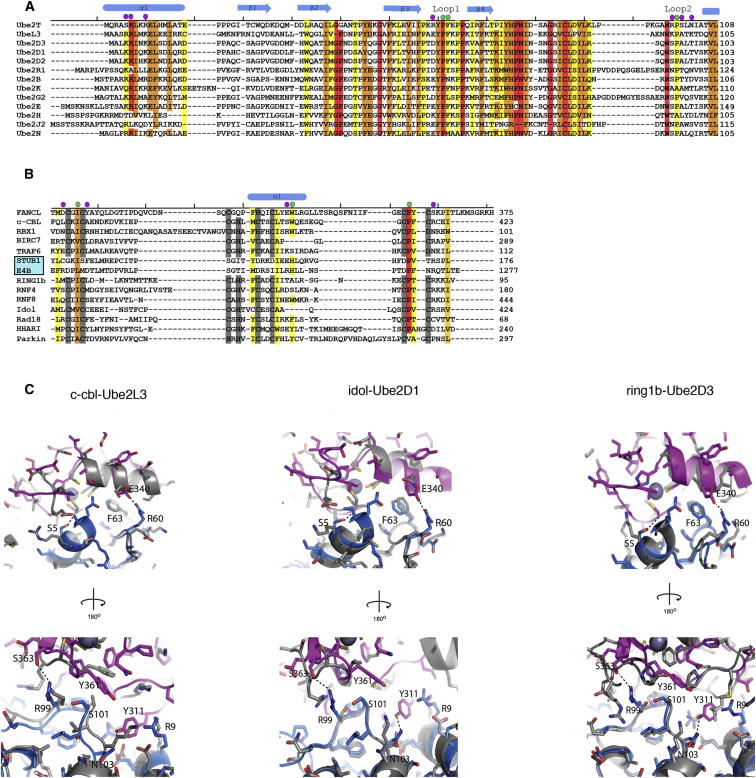
Structural Comparison of the FANCL RING Domain-Ube2t Complex with Other RING-E2 Complexes (A) A structure-based sequence alignment of E2s. PDB ID codes of E2s, as listed in the figure: 1FBV, 3RPG, 4AP4, 4AUQ, 3RZ3, 2YB6, 3K9O, 3H8K, 2Z5D, 2F4W, 3HCT, and 3BZH. (B) A structure-based sequence alignment of RING and Ubox domains. Ubox domains are highlighted by a cyan box. PDB ID codes used of RING and Ubox domains, as listed in the figure: 1FBV, 4F52, 4AUQ, 3HCT, 2C2V, 3LIZ, 3RPG, 4AP4, 4EPO, 2YHO, 2Y43, 4KBL, and 4K7D. Residues shaded in red to yellow colors indicate conserved residues, where red corresponds to strict conservation. Gray bars indicate zinc coordinating atoms. Green circles highlight residues involved in the hydrophobic interface between FANCL and Ube2T. Purple circles denote residues involved in hydrogen bonding and electrostatic interactions in the FANCL Ube2T interface. (C) Superpositions of the FANCL RING-Ube2T complex (colored pink and blue, respectively), with c-cbl RING-Ube2L3 complex (left) shaded gray (PDB ID code 1FBV), idol-Ube2D1 complex (middle) shaded gray (PDB ID code 2YHO), and ring1b-Ube2D3 complex (right) shaded gray (PDB ID code 3RPG). Numbered residues are the same as the FANCL RING-Ube2T complex, with dashed lines showing interactions.

**Figure 3:**
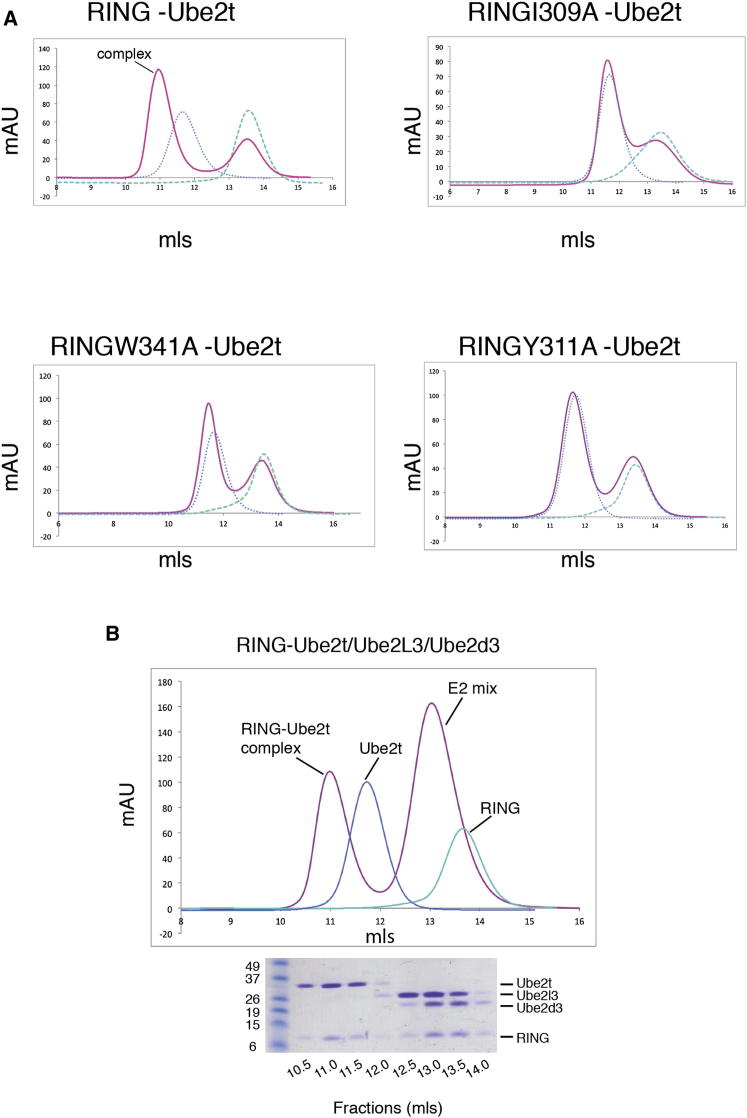
Conserved Hydrophobic RING Residues Are Required for Ube2T Binding and FANCL Selects Solely Ube2T In Vitro (A) Size-exclusion chromatogram profiles of wild-type (WT) or mutant RING domains (green dashed line) and WT Ube2T (blue dotted line) overlaid with profiles from binding experiments in which WT Ube2T has been incubated with WT or mutant RING domains (pink line) and subjected to size-exclusion chromatography. Binding was assessed by complex formation, which is indicated by a peak shift to the left labeled complex. (B) Size-exclusion chromatogram of FANCL RING domain incubated with an E2 mix consisting of Ube2T, Ube2D3, and Ube2L3 (pink line). Chromatograms of Ube2T (blue dotted line) and the RING domain (green dashed line) are also overlaid. A peak shift to the left is observed, indicating complex formation. SDS-PAGE gel of the fractions collected from the size-exclusion experiment and stained with Coomassie Brilliant Blue. The E2 gel bands found in the shifted peak were assessed by mass spectrometry for protein identification and confirmed as exclusively Ube2T. See also Figure S2.

Structure-based alignments reveal conservation of the residues attributable to the hydrophobic interface across RING domains and E2s (Figure 2). Based on our observations, we hypothesized that not only the conserved hydrophobic residues Ile309 and Trp341, but also the FANCL-specific Tyr311 revealed by our structure, are important for the FANCL-Ube2T interaction. To test this hypothesis, we purified single RING-point mutants Ile309Ala, Tyr311Ala, and Trp341Ala. In contrast to wild-type (WT) RING, each single-point mutant fails to form a complex with Ube2T (Figure 3A). Interestingly, our structure-based alignments reveal the residues involved in the electrostatic and hydrogen-bonding network observed in the FANCL-Ube2T interface are highly variable (Figures 2A and 2B). This suggests that these interactions are specific to this pair. Therefore, we assessed other E2s for their ability to bind FANCL. We tested Ube2L3, Ube2D3, Ube2L6, Ube2R1, Ube2K, Ube2H, and Ube2B, all of which possess the conserved hydrophobic interface residues (except Ube2B, which has an asparagine at position Phe63 of Ube2T) but are not conserved in the residues responsible for the electrostatic and hydrogen bonding network (Figure 2A). In contrast to Ube2T, none of the other E2s were competent to complex with FANCL using analytical size-exclusion chromatography (Figure S2A) and native gel shift assays (Figure S2B). In addition, Ube2T is unable to complex with another RING domain, Rbx1 (Figure S2A). Taken together, these results demonstrate the importance of the additional interactions for E2-E3 selectivity.

Although FANCL does not form a complex with other E2s, the conserved nature of the E2 UBC-fold and hydrophobic interface suggests the possibility that, in the absence of Ube2T, FANCL could function with another E2. To test this, we assayed the monoubiquitination of FLAG-FANCD2 by FANCL with various E2s (Figure S2C). In contrast to Ube2T (lane 2 of each blot), none of the other E2s were able to specifically monoubiquitinate FANCD2. The very promiscuous E2, Ube2D3 (Brzovic and Klevit, 2006) is capable of polyubiquitinating FANCD2 in the absence of FANCL (lane 5, Figure S2C). Importantly, the addition of FANCL (lane 6, Figure S2C) does not change the modification to a monoubiquitination event; it also does not enhance the amount of polyubiquitinated FANCD2. These results further support the observed promiscuity of Ube2D3 for lysines (Wenzel et al., 2011).

Our structural and biochemical analyses suggest that, in a cellular environment with multiple E2s present, FANCL will preferentially select Ube2T. In order to assess FANCL’s E2 selectivity, we incubated the FANCL RING domain with equimolar amounts of different E2s, Ube2T, Ube2D3, and Ube2L3 and assessed the ability of FANCL to select Ube2T by analytical size-exclusion chromatography (Figure 3B). Indeed, FANCL exclusively formed a complex with Ube2T, as confirmed by SDS-PAGE analysis of collected fractions and protein identification by mass spectrometry (Figure 3B).

**Figure 4:**
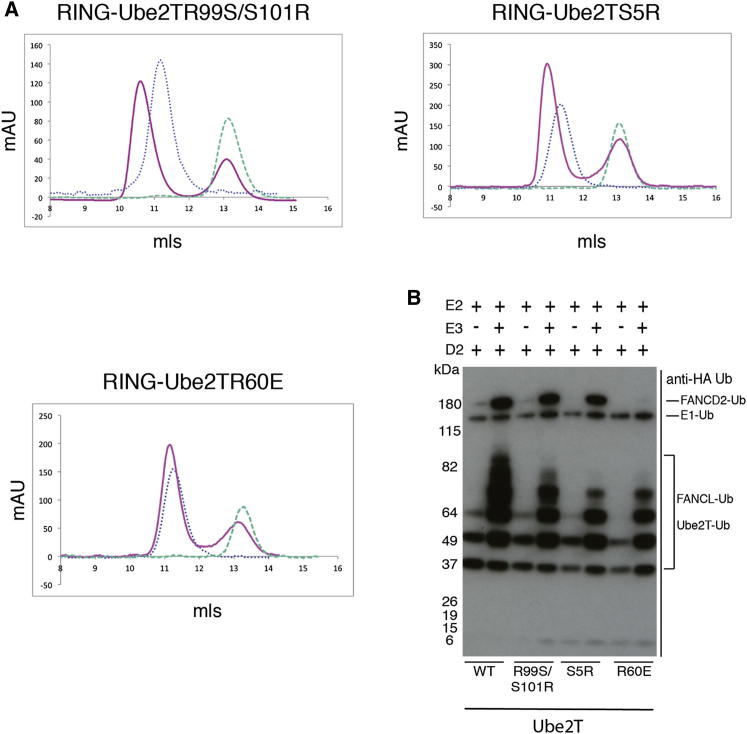
Additional Residues of Ube2T Are Required for Binding the RING Domain of FANCL (A) Size-exclusion chromatogram profiles of wild-type (WT) or mutant Ube2T (blue) dotted line and WT RING domains (green dashed line) overlain with profiles from binding experiments in which WT RING domain has been incubated with WT or mutant Ube2T (pink line) and subjected to size-exclusion chromatography. Binding was assessed by complex formation indicated by a peak shift to the left. (B) An anti-HA-Ub western blot of in vitro monoubiquitination assays to assess monoubiquitination of FLAG-FANCD2 by FANCL in collaboration with different WT Ube2T and Ube2T mutants (Ube2TArg99Ser/Ser101Arg, Ube2TSer5Arg, and Ube2TArg60Glu). Lanes 2, 4, and 6 show the monoubiquitination of FLAG-FANCD2 when WT Ube2T, Ube2T Arg99Ser/Ser101Arg, and Ube2TSer5Arg are paired with FANCL. Monoubiquitination is not observed for with the Ube2TArg60Glu mutant is used (lane 8).

It is clear from our results that FANCL preferentially selects Ube2T. Although FANCL extends its hydrophobic surface for interaction with Ube2T by Tyr311, the corresponding Ube2T-interacting residues Arg6 and Arg9 are conserved in some of the E2s we have tested for FANCL binding and function (e.g., Ube2K, Figure 2A). Therefore, the selectivity of FANCL for Ube2T must be attributed to the electrostatic and hydrogen bonding interactions, which are highly variable among the E2s (Figure 2A). In order to test this hypothesis, we incubated purified mutants of Ube2T Ser5Arg, Arg60Glu, and Arg99Ser/Ser101Arg with the wild-type FANCL RING domain and assessed interaction by size-exclusion chromatography. Only the Arg60Glu mutant of Ube2T is unable to bind the FANCL RING domain (Figure 4A). Consistent with the binding profile of Ube2T mutants, Ser5Arg-Ube2T, Arg99Ser/Ser101Arg-Ube2T, and wild-type Ube2T all support FANCL-dependent monoubiquitination of FANCD2 (Figure 4B). By contrast, Arg60Glu-Ube2T is unable to either bind FANCL or facilitate FANCD2 monoubiquitination (lane 8, Figure 4B). We therefore conclude that the positive selector in Ube2T for FANCL is Arg60, which forms a salt bridge with Glu340 of FANCL (Figure 1D) and is required for FANCL-Ube2T-mediated monoubiquitination of FANCD2.

The dearth of specific E3-E2 structures has hampered the understanding of how E3s select their E2s. Our structure of FANCL-Ube2T reveals a specific extensive electrostatic and hydrogen bonding network surrounding a conserved hydrophobic interaction. In particular, Tyr311 of FANCL, which is a highly variable residue in other E3s, acts like a key in a lock, pi stacking between Arg6 and Arg9 of Ube2T. Additionally the nonconserved Asn103 of Ube2T further anchors Tyr311 of FANCL into position. Ser5 of Ube2T acts as a negative selectivity factor: as in other E2s it is a much larger, bulkier residue and is typically arginine or lysine. These residues would result in loss of the hydrogen bond that occurs between Ser5 of Ube2T and the main chain oxygen of Cys310 of FANCL and potentially clash with FANCL residues Cys310 or Ala312 and/or the main chain of Gln314. Importantly, we have identified Arg60 of Ube2T as the positive selector for FANCL (Figures 1D, 2A, and 4). In other E2s, the equivalent position frequently has the opposite charge (Glu in Ube2L3, Asp in the Ube2D family, and Glu in Ube2B). Ube2E1 and Ube2E2 have 97% sequence identity and share common E3s but also have distinct E3 partners. The residue equivalent to Arg60 in Ube2T is predicted to be important for this distinction in a recent proteome-scale modeling study (Kar et al., 2012). Our study provides experimental support for the importance of this position in a more divergent E2. Arg60 of Ube2T forms a salt bridge with Glu340 of FANCL (Figure 1D). In other E3s, the equivalent residue is poorly conserved (Figure 2B).

Our structural and biochemical data can be used to refine algorithms for predicting E3-E2 pairs and provide a druggable platform for the development of chemotherapeutics.

## Experimental Procedures

### Protein Expression and Purification

To generate the RING-linker-Ube2T fusion construct, we cloned human FANCL RING domain (residues 289–375) from synthetic human FANCL DNA (GeneArt) codon-optimized for *Escherichia coli* expression and inserted N terminally to Ube2T encoded in a vector containing a N-terminal 6xHis-Smt3 tag, by restriction-free cloning (RF) (van den Ent and Löwe, 2006). A 14 amino acid (TGSTGSTETGYTQG) linker was inserted between the C-terminal of the RING domain and N-terminal of Ube2T (Pellegrini et al., 2002) by Phusion site-directed mutagenesis. Human Ube2T, Mouse Rbx1, and *Xenopus tropicalis* FANCL were cloned from I.M.A.G.E clones (Geneservice) into a vector containing a 6x His-Smt3 tag by RF methods. The Human FANCL RING domain (residues 289–375) was cloned from the synthetic human FANCL DNA as described above. Human Ube2D3 (UbcH5c), Ube2L3 (UbcH7), Ube2K, Ube2B, and Ube2R1 were cloned from I.M.A.G.E. clones (Geneservice) and inserted into the pDEST17 (Invitrogen) and pET RSF vectors containing an N-terminal 6xHis tag and a TEV cleavage site. Ube2H was purchased as a synthetic gene in expression vector pJ441 containing a 6xHis tag and a TEV cleavage site from DNA 2.0. Proteins were expressed in *E. coli* BL21 cells (Invitrogen) or Rosetta (DE3) cells (Millipore) in the case of *X. tropicalis* FANCL, Ube2K, and Ube2R1. Cells were cultured in Lysogeny broth (LB) supplemented with antibiotics and 0.5 mM ZnCl_2_ for proteins with a RING domain, at 37°C. Once OD_600_ had reached 0.6, protein expression was induced by the addition of 250 μM isopropyl-1-thio-β-d-galactopyranoside (IPTG) (500 μM in the case of the E2s). Cells were cultured overnight at 16°C and harvested the following day by centrifugation. Harvested cells were lysed by sonication of 4× 10s bursts on ice, in buffer containing 0.5 M NaCl, 0.1 M Tris (pH 8), 0.02 M Imidazole, and 250 μM tris(carboxyethyl)phosphine (TCEP). Cell debris was removed by centrifugation at 32,000 × *g*. Supernatants were added to equilibrated Ni-NTA Agarose (QIAGEN) and incubated on a roller for 1 hr at 4°C. 6xHis-Smt3 tags were removed overnight at 4°C by Ulp1 protease at a w/w ratio of 1:15, Ulp1:protein. *Xenopus tropicalis* FANCL was flash frozen at 0.5 mg/ml and stored at −80°C. Ube2D3, Ube2L3, Ube2K, Ube2B, Ube2R1, and Ube2H were eluted from agarose, and their 6xHis tags were removed with His-TEV protease, added at w/w ratio of 1:15, His-TEV protease:protein, as an overnight dialysis step at 4°C. Samples were concentrated the following day and loaded onto either a Superdex 75 or Superdex 200 column. Purified fractions were pooled and concentrated before flash freezing and stored at −80°C.

### *Xenopus laevis*

*Xenopus laevis* FANCD2 plasmid was a kind gift from P. Knipscheer and J. C. Walter. We modified it to contain a N-terminal FLAG tag and prepared it as previously described (Knipscheer et al., 2009).

Mouse His-UBE1 was a kind gift from K. Iwai (Sato et al., 2008). It was expressed in sf9 cells cultured at 27°C in sf-900 serum-free media (GIBCO) supplemented with antibiotics. Cells were harvested 3 days postinfection and lysed by sonication, 2× 5 s bursts in buffer containing 0.05 M Tris (pH 8), 0.1 M NaCl, 0.5 mM PMSF, and 1 mM EDTA. Cell debris was removed by high-speed centrifugation, 32,000 × *g*, and supernatants were added to equilibrated Ni-NTA Agarose. The His-UBE1 was then eluted from the agarose, concentrated, and applied to a Superdex 200 column. Purified His-UBE1was then flash frozen and stored at −80°C.

Human RING, Ube2T, and *Xenopus laevis* FANCD2 mutants were generated using site-directed mutagenesis and expressed as WT proteins.

### Crystallization and Structure Determination

The RING-Ube2T fusion crystals were grown using a final concentration of 11.7 mg/ml by sitting drop vapor diffusion at 4°C in spacegroup P4_3_2_1_2 with cell dimensions of a = 109.3 Å, b = 109.3 Å, c = 117.7 Å, α = 90°, β = 90°, and γ = 90°. Crystallization conditions were 1.6 M ammonium sulfate, 0.1 M NaCl, 0.1 M HEPES (pH 7.5). Crystals were cryoprotected with 20% glycerol and cryocooled in liquid nitrogen. Data were collected at Diamond Light Source on the microfocus I24 beamline at 0.96 Å wavelength. Data were processed using D^∗^trek (Pflugrath, 1999), showing diffraction to 2.25 Å resolution. An estimated solvent content of 52.7% suggested two copies of the polypeptide chain in the asymmetric unit (ASU). Phases were generated by molecular replacement using the program Phaser (McCoy et al., 2007) with Ube2T (residues 1–154, PDB ID code 1YH2) and *Drosophila* FANCL (residues 312–371, 3K1L) (Cole et al., 2010) as search models. The model was refined iteratively using phenix.refine (Afonine et al., 2005) and manual model building using Coot (Emsley and Cowtan, 2004). Data were cut off to 2.4 Å resolution, and the last 400 images were omitted because of radiation damage. Omit maps were generated to check for model bias. The final model’s stereochemistry and geometry was checked with MolProbity (Davis et al., 2004) analysis (Table 1) and for favored regions of the Ramachandran plot (97.52%). Table 1 summarizes data collection and refinement statistics. The ASU contains four chains, two chains of Ube2T (A and B), and two chains of FANCL RING domain (X and Y). In both chains of Ube2T, electron density was not observed for the C-terminal (residues 154–197), and for chain B, the loop region from residue 27–32 was not modeled because of poor electron density. For chain Y of the RING domain, residues 352 and 353 were not modeled because of a symmetry contact leading to poor electron density. Additionally, there was no observed electron density for the majority of the fusion linker. The RING domain, chain X interacts with chain A of Ube2T, and the RING domain chain Y interacts with chain B of Ube2T. Also, Arg9 in chain A is not seen in the interaction interface because of its displacement by a symmetry molecule.

### Structural Analysis

All structural analyses were carried out using PyMOL (Delano, 2002). Structural alignments were produced and manually adjusted using MegAlign software. The structural interface was calculated using PISA.

### Analytical Size-Exclusion Chromatography

Interactions between RING domains and E2s were assessed by analytical size-exclusion chromatography, as described previously (Hodson et al., 2011). Briefly, interactions were incubated in buffer containing 0.1 M NaCl, 0.1 M Tris (pH 8), and 250 μM TCEP in a total volume of 500 μl and left on ice for 1 hr. Samples were loaded onto a Superdex 75 10/300 column (GE Healthcare), and 0.5 ml fractions were collected. Fractions were analyzed on 12% SDS-PAGE gels.

### Native Gel Assays

Interactions between FANCL RING domain and E2s were assessed by native gel assays. Excess FANCL RING domain (∼140 μM) was incubated with 80–100 μM of E2 and incubated in buffer containing 0.1 M NaCl, 0.1 M Tris (pH 8), and 250 μM TCEP in a total volume of 10 μl and left on ice for 10 min. A 10 μl of loading buffer containing 10% glycerol was added to the samples, and the samples were analyzed on 4%–12% native PAGE gels (Invitrogen). Gels were stained with SimplyBlue SafeStain (Invitrogen).

### FANCD2 Monoubiquitination Assay

The ability of FANCL to monoubiquitinate FANCD2 using different E2s was assessed by an in vitro monoubiquitination assay. Reaction volumes of 25 μl contained 17 nM His-UBE1, 0.64 μM E2, 1.86 μM *Xenopus tropicalis* FANCL, 4.2 μM HA-Ub (Boston Biochem), 0.5 μM *Xenopus laevis* FLAG-FANCD2 or FLAG-FANCD2K562R, and reaction buffer: 50 mM Tris (pH 7.5), 100 mM KCl_2_, 2 mM MgCl_2_, 0.5 mM DTT, and 2 mM ATP. Ube2D3, Ube2L6, and Ube2N/Ube2V1 were purchased from Boston Biochem. Reactions were left for 1.5 hr at room temperature. A 25 μl volume of LDS buffer (Invitrogen) containing BME was used to terminate reactions. Samples were loaded onto a 4%–12% SDS-PAGE gel and subjected to western blotting. Anti-HA antibody, raised against HA peptide (Pettinghill Technology), anti-FLAG antibody (Abcam), and anti-FANCD2 (Abcam), was used to probe for FLAG-FANCD2 monoubiquitination.

## Figures and Tables

**Table 1:** Data Collection and Refinement Statistics

Data Collection	
Beamline	I24 (DLS)
Wavelength (Å)	0.96
Resolution range (Å)	46.8–2.4 (2.5–2.4)
Space group	P43212
Cell dimensions (Å)	a = 109.3, b = 109.3, c = 117.7
Cell dimensions (°)	α = 90, β = 90, γ = 90
Unique reflections	28,423
Multiplicity	6.8 (7.2)
Completeness (%)	100 (100)
Rmeas (%), Rpim (%), CC1/2	13.8 (81.1), 5.6 (45.1), 0.99 (0.47)
<I/σ>	5.7 (1.2)

**Refinement**	

PDB ID code	4CCG
R_work_ / R_free_	21.2/24.8
No. of non-H atoms	3,744
Mean B value (Å^2^)	61.6
Rmsd bond lengths (Å)	0.004
Rmsd bond angles (°)	0.670
MolProbity clashscore	2.52

Highest-resolution shell is given in parentheses. Rmsd, root-mean-square deviation.

## Accession Numbers

The Protein Data Bank accession number for the atomic coordinates reported in this paper is 4CCG.
