# Supplementary material for: Structure of the Human FANCL RING-Ube2T Complex Reveals Determinants of Cognate E3-E2 Selection
Source: Structure. 2014 Feb 4;22(2):337–44. doi: 10.1016/j.str.2013.12.004 (PMC3979106; doi:10.1016/j.str.2013.12.004)
Supplement: Document S1. Figures S1 and S2 [file mmc1.pdf]

Structure, Volume *22*

Supplemental Information

Structure of the Human FANCL RING-Ube2T Complex Reveals Determinants of Cognate E3-E2 Selection

Charlotte Hodson, Andrew Purkiss, Jennifer Anne Miles, and Helen Walden

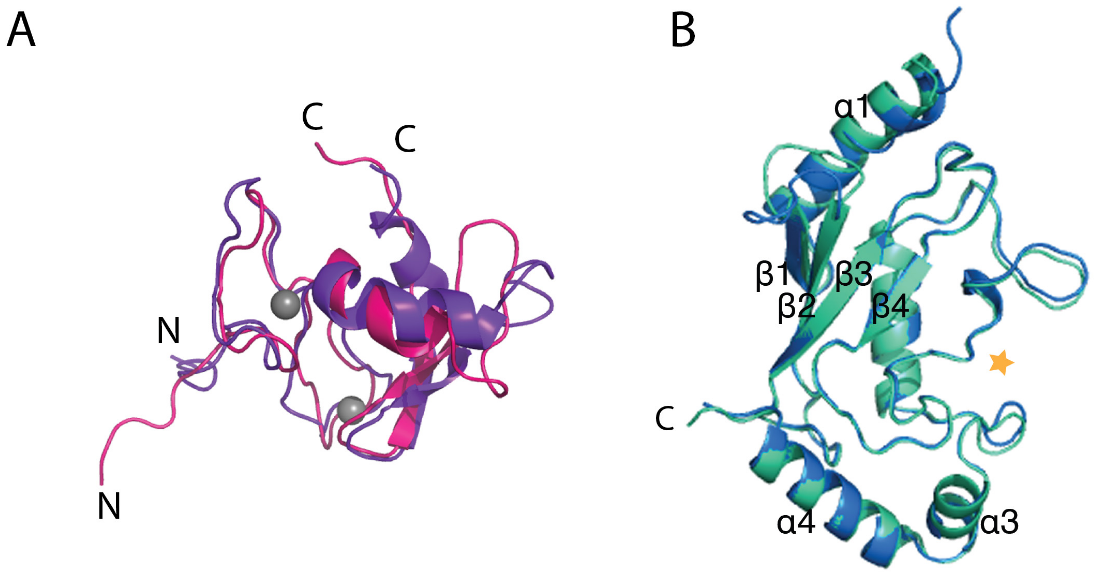

Figure S1 related to Figure 1 Overall Structure of FANCL-Ube2T complex

A) Overlay of human (magenta) and *Drosophil*a (purple, PDB code 3K1L) FANCL RING domains. **B)** Superposition of unbound Ube2T (green, PDB code 1YH2) with Ube2T bound to the FANCL RING domain (blue). A gold star represents the position of the catalytic cysteine.

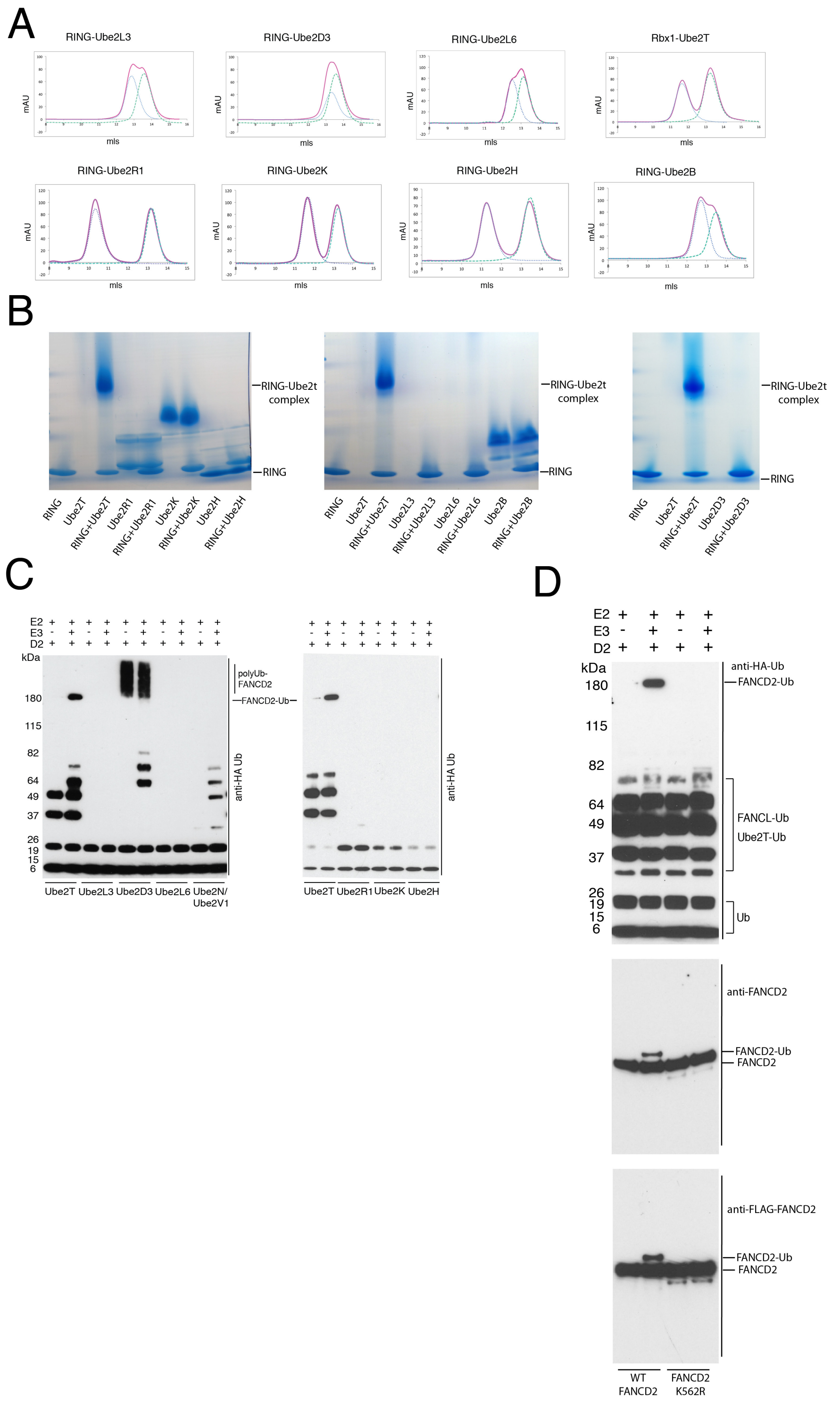

Figure S2 related to Figure 3 FANCL functions solely with Ube2T for FANCD2 monoubiquitination *in vitro*

A) Size exclusion chromatogram profiles of FANCL or Rbx1 RING domain (green dashed line) and different E2s (blue dotted line) overlaid with profiles from binding

experiments where the RING domain has been incubated with either Ube2D3, Ube2L3, Ube2L6, Ube2R1, Ube2K, Ube2H or Ube2B (pink line) and subjected to size exclusion chromatography. Binding was assessed by complex formation indicated by a peak shift to the left, of which no peak shifts were observed. **B)** Interactions between human FANCL RING domain and E2s assessed by native gel electrophoresis. **C**) Anti-HA-Ub western blots of *in vitro* monoubiquitination assays to assess monoubiquitination of FLAG-FANCD2 by FANCL in collaboration with different E2s. Lane 2 for both western blots shows the monoubiquitination of FLAG-FANCD2 when Ube2T is paired with FANCL. Monoubiquitination is not observed for other E2-FANCL pairings (lanes 4, 6, 8 and 10 both western blots). Lower bands correspond to E2 and E3 autoubiquitination, and HA-ubiquitin. **D)** Western blots of an *in vitro* monoubiquitination assay showing monoubiquitination of FLAG-FANCD2 (lane 2) using anti-HA Ubiquitin (upper blot), anti-FANCD2 (middle blot) and anti-FLAG (lower blot). Lanes 3 and 4 use FLAG-FANCD2 K562R mutant, which is not monoubiquitinated.
